# Age-related upregulation of dense core vesicles in the central inferior colliculus

**DOI:** 10.3389/fncel.2024.1396387

**Published:** 2024-05-01

**Authors:** Jeffrey G. Mellott, Syllissa Duncan, Justine Busby, Laila S. Almassri, Alexa Wawrzyniak, Milena C. Iafrate, Andrew P. Ohl, Elizabeth A. Slabinski, Abigail M. Beaver, Diana Albaba, Brenda Vega, Amir M. Mafi, Morgan Buerke, Nick J. Tokar, Jesse W. Young

**Affiliations:** ^1^Department of Anatomy and Neurobiology, Northeast Ohio Medical University, Rootstown, OH, United States; ^2^University Hospitals Hearing Research Center, Northeast Ohio Medical University, Rootstown, OH, United States; ^3^The Ohio State University College of Medicine, Columbus, OH, United States; ^4^Department of Psychology, Louisiana State University, Baton Rouge, LA, United States

**Keywords:** inferior colliculus, GABA, synapse, aging, dense core vesicles

## Abstract

Presbycusis is one of the most prevalent disabilities in aged populations of industrialized countries. As we age less excitation reaches the central auditory system from the periphery. To compensate, the central auditory system [e.g., the inferior colliculus (IC)], downregulates GABAergic inhibition to maintain homeostatic balance. However, the continued downregulation of GABA in the IC causes a disruption in temporal precision related to presbycusis. Many studies of age-related changes to neurotransmission in the IC have therefore focused on GABAergic systems. However, we have discovered that dense core vesicles (DCVs) are significantly upregulated with age in the IC. DCVs can carry neuropeptides, co-transmitters, neurotrophic factors, and proteins destined for the presynaptic zone to participate in synaptogenesis. We used immuno transmission electron microscopy across four age groups (3-month; 19-month; 24-month; and 28-month) of Fisher Brown Norway rats to examine the ultrastructure of DCVs in the IC. Tissue was stained post-embedding for GABA immunoreactivity. DCVs were characterized by diameter and by the neurochemical profile (GABAergic/non-GABAergic) of their location (bouton, axon, soma, and dendrite). Our data was collected across the dorsolateral to ventromedial axis of the central IC. After quantification, we had three primary findings. First, the age-related increase of DCVs occurred most robustly in non-GABAergic dendrites in the middle and low frequency regions of the central IC during middle age. Second, the likelihood of a bouton having more than one DCV increased with age. Lastly, although there was an age-related loss of terminals throughout the IC, the proportion of terminals that contained at least one DCV did not decline. We interpret this finding to mean that terminals carrying proteins packaged in DCVs are spared with age. Several recent studies have demonstrated a role for neuropeptides in the IC in defining cell types and regulating inhibitory and excitatory neurotransmission. Given the age-related increase of DCVs in the IC, it will be critical that future studies determine whether (1) specific neuropeptides are altered with age in the IC and (2) if these neuropeptides contribute to the loss of inhibition and/or increase of excitability that occurs during presbycusis and tinnitus.

## 1 Introduction

The auditory system plays a crucial role in our everyday lives by enabling communication, sound localization, and information processing. Conversely, auditory system dysfunction leads to impaired communication, difficulty participating in noisy social environments, and an overall decrease in quality of life. As the aging population continues to grow, there is an increased demand for understanding the mechanisms underlying hearing loss, which affects nearly three-quarters of individuals aged 70 years or older (Goman and Lin, 2016). The functional decline of the auditory system with age often begins with the degradation of the peripheral auditory system, which leads to a decrease in excitation sent to the central auditory system (Knipper et al., 2022; Rumschlag et al., 2022). The central auditory system compensates for this decreased excitation by downregulating GABAergic inhibition; this is believed to be an attempt at restoring homeostatic levels of activity (Caspary et al., 2008; Richardson et al., 2012; Caspary and Llano, 2018). Unfortunately the gradual loss of GABA into old age leads to functional deficits and increased central neural gain (Caspary et al., 2008; Auerbach et al., 2014, 2019). Age-related downregulation of GABAergic inhibition is well documented in the inferior colliculus (IC), an auditory midbrain structure that serves as the hub of the central auditory system (Wenstrup, 2005; Caspary et al., 2008; Syka, 2020). The IC is organized into three subdivisions [central (ICc), lateral cortex (IClc), and dorsal cortex (ICd)] and processes information from multiple ascending and descending auditory projections (see reviews: Oliver, 2005; Syka, 2020). Neurons and inputs within the lemniscal ICc are arranged into isofrequency lamina, which are organized tonotopically along the ventromedial (high frequencies) to dorsolateral (low frequencies) axis (Oliver and Morest, 1984; Oliver, 2005; Malmierca et al., 2008; Syka, 2020).

Considering that mammalian presbycusis is frequency specific, it is important to understand age-related molecular and ultrastructural changes in relation to the tonotopic axis (Walton et al., 1998; Cai et al., 2018; Parthasarathy et al., 2018; Koehler et al., 2023). In our ongoing investigations of the aging synaptic ultrastructure in the IC, we observed a dramatic increase of dense core vesicles (DCVs) in the aged IC. In the current study, we quantify and characterize this increase in DCVs. DCVs are membrane-bound organelles that contain dense granular cores; they are one of two types of organelles that secrete chemical signals throughout the nervous system, with classic clear synaptic vesicles being the other (Sorra et al., 2006; Persoon et al., 2018). Unlike synaptic vesicles which are found in large pools at active zones and carry classic neurotransmitters, DCVs are rarer, and they tend to package biogenic amines, neuromodulators, presynaptic machinery, neuropeptides, neurotrophic factors, and hormones (Peters et al., 1991; Michael et al., 2006; Sorra et al., 2006; Merighi, 2018). A number of important neuromodulators involved in auditory midbrain function are transported by DCVs, including serotonin, neuropeptide Y (NPY), vasoactive intestinal peptide (VIP), brain-derived neurotrophic factor (BDNF), and fibroblast growth factors (Pelletier et al., 1981; Sato et al., 2001; Dieni et al., 2012; Schofield and Hurley, 2018; Goyer et al., 2019; Silveira et al., 2020, 2023). DCVs are transported via both dendritic and axonal trafficking, where microtubule dependent kinesin-1 motors (KIF1) and motor dynein mediate trafficking into dendrites and axons (Zheng et al., 2008; Lipka et al., 2016). It is unclear whether DCVs are preferentially released at axons or dendrites (Kennedy and Ehlers, 2011; Persoon et al., 2018). To our knowledge, very little is understood about the populations of DCVs in the auditory midbrain, including what proteins they contain and their trafficking patterns.

The goal of the present study is to determine whether DCVs are upregulated in the aging ICc. Specifically, we examined three tissue blocks taken across the ventromedial-dorsolateral ICc axis. For the sake of conciseness, alignment with our previous studies, and convention as the tonotopic axis is well established, we refer to the ventromedial-most ICc block as representing the high frequency region, the middle block as representing the middle frequency region, and dorsolateral-most block as representing lower frequencies. We used immuno transmission electron microscopy to analyze DCVs in GABAergic and non-GABAergic boutons, axons, and dendrites of Fischer Brown Norway (FBN) rats across four (3–4 months “young”; 19–20 months “early middle-age”; 24 months “late middle-age”; and 28–29 months “old”) age groups. We discovered that: (1) DCVs were markedly increased in non-GABAergic dendrites during middle age. Many of these DCVs were located near postsynaptic densities. (2) The probability of a GABAergic or non-GABAergic bouton having more than one DCV increased with age. (3) Despite the age-related loss of boutons throughout the IC, the percentage of non-GABAergic and GABAergic boutons that had at least one DCV did not decline with age. We conclude that synapses lost in the IC in aging may not be synapses that co-release neuropeptides. Ultimately, it appears that the many possible proteins packaged by DCVs may have a significant role in the processing of acoustic signals in the aging IC.

## 2 Materials and methods

### 2.1 Animals

All procedures were conducted in accordance with the Northeast Ohio Medical University Institutional Animal Care and Use Committee and NIH guidelines. Results are described from 20 male FBN rats [National Institute of Aging; Bethesda, MD, USA; RRID:SCR_007317 (housed by Charles River Laboratories, Wilmington, MA, USA)] across four age groups (5 animals per age group): 3–4 months “young”; 19–20 months “early middle-age”; 24 months “late middle-age”; and 28–29 months “old” (Tables 1–3). For a details regarding ambient sound levels, please see Cai et al. (2018). Efforts were made to minimize the number of animals and their suffering.

**TABLE 1:** Summary of ICc DCV in GABA-negative profiles.

	# of animals	Area analyzed (μm^2^)	# of DCV	Average diameter (nm)	# of DCV in bouton	# of DCV in axon	# of DCV in dendrite
**3–4 month**							
High frequency	5	16,000 (3,200/animal)	175	69.2	75	16	84
Middle frequency	5	16,000 (3,200/animal)	184	66.2	89	11	83
Low frequency	5	16,000 (3,200/animal)	194	69.2	100	13	81
**19–20 month**							
High frequency	5	16,000 (3,200/animal)	356	65.3	162	9	185
Middle frequency	5	16,000 (3,200/animal)	311	69.7	113	20	188
Low frequency	5	16,000 (3,200/animal)	267	70.5	110	23	142
**24 month**							
High frequency	5	16,000 (3,200/animal)	438	68.8	162	27	244
Middle frequency	5	16,000 (3,200/animal)	428	71.1	160	39	225
Low frequency	5	16,000 (3,200/animal)	382	70.9	133	23	226
**28 month**							
High frequency	5	16,000 (3,200/animal)	399	62.4	147	24	222
Middle frequency	5	16,000 (3,200/animal)	351	65.2	146	14	190
Low frequency	5	16,000 (3,200/animal)	326	64.7	133	26	165
**Summary of ICc DCV in GABAergic profiles**							
**3–4 month**							
High frequency	5	16,000 (3,200/animal)	85	69.8	49	9	24
Middle frequency	5	16,000 (3,200/animal)	110	70.9	42	18	44
Low frequency	5	16,000 (3,200/animal)	97	74.2	62	10	24
**19–20 month**							
High frequency	5	16,000 (3,200/animal)	110	65.3	64	7	39
Middle frequency	5	16,000 (3,200/animal)	116	71.6	65	9	34
Low frequency	5	16,000 (3,200/animal)	102	67.4	50	12	34
**24 month**							
High frequency	5	16,000 (3,200/animal)	163	71	68	9	82
Middle frequency	5	16,000 (3,200/animal)	165	73.8	86	14	61
Low frequency	5	16,000 (3,200/animal)	148	69.3	86	9	50
**28 month**							
High frequency	5	16,000 (3,200/animal)	188	61.2	76	18	84
Middle frequency	5	16,000 (3,200/animal)	158	68.2	89	9	51
Low frequency	5	16,000 (3,200/animal)	125	65.1	53	19	39

**TABLE 2:** Excitatory bouton breakdown.

	Total boutons	Bouton loss from 3 months	Total DCVs	# of boutons w/1 DCV	# of boutons w/2 DCV	# of boutons w/3 DCV	# of boutons w/4 DCV	# of boutons w/5 DCV	# of boutons w/>5 DCV	# of boutons w/at least 1 DCV	% boutons w/at least 1 DCV
**3–4 months**											
**High frequency**											
B72; R23	224		13	8	1	1	–	–	–	10	
B96; R41	207		9	5	2	–	–	–	–	7	
B123; R42	202		10	7	–	1	–	–	–	8	
B129; R116	194		22	12	3	–	1	–	–	16	
B132; R117	240		21	10	4	1	–	–	–	15	
Total	1,067	N/A	75	42	10	3	1	0	0	56	5.24%
**Middle frequency**											
B73; R23	208		13	4	3	1	–	–	–	8	
B97; R41	215		12	10	2	–	–	–	–	12	
B124; R42	219		19	11	1	2	–	–	–	14	
B130; R116	185		25	13	3	2	–	–	–	18	
B133; R117	204		20	14	1	–	1	–	–	16	
Total	1,031	N/A	89	52	10	5	1	0	0	68	6.60%
**Low frequency**											
B74; R23	217		17	13	–	–	1	–	–	14	
B98; R41	225		8	6	1	–	–	–	–	7	
B125; R42	192		21	10	1	3	–	–	–	14	
B131; R116	231		36	21	3	3	–	–	–	27	
B134; R117	237		18	7	4	1	–	–	–	12	
Total	1,096	N/A	100	57	9	7	1	0	0	74	6.80%
**19–20 months**											
**High frequency**											
B78; R63	184		41	15	5	4	1	–	–	25	
B81; R98	197		24	15	1	1	1	–	–	18	
B84; R99	204		33	14	3	3	1	–	–	21	
B105; R84	187		35	16	4	2	–	1	–	23	
B142; R96	209		29	14	3	3	–	–	–	23	
Total	981	9.10%	162	74	16	13	3	1	0	110	11.20%
**Middle frequency**											
B79; R63	186		23	5	6	2	–	–	–	13	
B82; R98	196		19	7	2	1	–	1	–	11	
B103; R83	218		39	8	5	2	3	–	–	18	
B106; R84	154		5	5	–	–	–	–	–	5	
B141; R96	201		27	15	6	–	–	–	–	21	
Total	955	7.40%	113	40	19	5	3	1	0	68	7.12%
**Low frequency**											
B80; R63	202		36	9	4	4	1	1	–	19	
B86; R99	207		27	5	4	2	2	–	–	13	
B104; R83	179		20	10	5	–	–	–	–	15	
B107; R84	200		11	6	1	1	–	–	–	8	
B143; R96	221		16	9	2	1	–	–	–	12	
Total	1,009	8%	110	39	16	8	3	1	0	67	6.60%
**24 months**											
**High frequency**											
B111; R101	158		23	5	3	–	3	–	–	11	
B114; R102	156		28	9	4	2	–	1	–	16	
B117; R100	170		27	11	4	–	2	–	–	17	
B135; R109	143		25	6	2	5	–	–	–	13	
B138; R110	187		59	16	6	4	3	–	1 (7)	29	
Total	814	23.80%	162	47	19	11	8	1	1	86	11%
**Middle frequency**											
B112; R101	202		25	4	5	2	–	1	–	12	
B115; R102	179		53	25	2	2	2	2	–	33	
B118; R100	132		31	5	2	2	4	–	–	13	
B121; R108	166		19	10	–	3	–	–	–	13	
B136; R109	137		32	7	2	5	–	–	1 (6)	15	
Total	816	20.90%	160	51	11	14	6	3	1	86	10.50%
**Low frequency**											
B113; R101	167		19	9	1	1	–	1	–	12	
B116; R102	217		47	10	8	3	3	–	–	24	
B119; R100	176		7	5	1	–	–	–	–	6	
B122; R108	203		36	4	4	5	1	1	–	15	
B137; R109	160		24	12	3	2	–	–	–	17	
Total	923	15.80%	133	40	17	11	4	2	0	74	8.02%
**28–29 months**											
**High frequency**											
B87; R29	134		12	6	1	–	1	–	–	8	
B90; R46	154		48	5	6	7	–	2	–	20	
B93; R79	147		11	8	–	1	–	–	–	9	
B99; R45	134		33	7	7	4	–	–	–	18	
B126; R47	181		43	9	9	1	2	1	–	22	
Total	750	29.70%	147	35	23	13	3	3	0	77	10.30%
**Middle frequency**											
B88; R99	156		10	6	2	–	–	–	–	8	
B91; R46	173		29	5	3	4	–	–	1 (6)	13	
B94; R79	150		36	13	4	2	1	1	–	21	
B100; R45	158		42	4	4	5	2	–	1 (7)	16	
B127; R47	138		29	15	5	–	1	–	–	21	
Total	775	24.80%	146	43	18	11	4	1	2	79	10.20%
**Low frequency**											
B89; R99	167		12	5	2	1	–	–	–	8	
B92; R46	190		44	11	1	2	5	1		20	
B95; R79	189		34	12	4	2	2	–		20	
B101; R45	180		27	7	6	3	–	1	–	17	
B128; R47	177		16	14	1	–	–	–	–	15	
Total	903	17.60%	133	49	14	8	7	2	0	80	8.70%

“BXX” designates the block number. “RXX” designates the animal.

**TABLE 3:** GABAergic bouton breakdown.

	Total boutons	Bouton loss from 3 months	Total DCVs	# of boutons w/1 DCV	# of boutons w/2 DCV	# of boutons w/3 DCV	# of boutons w/4 DCV	# of boutons w/5 DCV	# of boutons w/>5 DCV	# of boutons w/at least 1 DCV	% of boutons w/at least 1 DCV
**3–4 months**											
**High frequency**											
B72; R23	188		8	6	2	–	–	–	–	8	
B96; R41	216		6	3	–	1	–	–	–	4	
B123; R42	180		7	4	–	1	–	–	–	5	
B129; R116	212		14	3	4	1	–	–	–	8	
B132; R117	219		14	7	2	1	–	–	–	10	
Total	1,015	N/A	49	23	8	4	0	0	0	35	3.50%
**Middle frequency**											
B73; R23	187		4	4	–	–	–	–	–	4	
B97; R41	170		7	5	1	–	–	–	–	6	
B124; R42	159		13	5	–	1	–	–	–	6	
B130; R116	208		14	3	2	1	1	–	–	7	
B133; R117	193		4	4	–	–	–	–	–	4	
Total	917	N/A	42	21	3	2	1	0	0	27	2.90%
**Low frequency**											
B74; R23	238		10	8	1	–	–	–	–	9	
B98; R41	204		8	7	1	–	–	–	–	8	
B125; R42	164		15	10	1	1	–	–	–	12	
B131; R116	172		21	11	5	–	–	–	–	16	
B134; R117	209		8	6	1	–	–	–	–	7	
Total	987	N/A	62	42	9	1	0	0	0	52	5.30%
**19–20 months**											
**High frequency**											
B78; R63	155		25	8	4	3	–	–	–	15	
B81; R98	171		13	6	–	1	1	–	–	8	
B84; R99	201		14	5	3	1	–	–	–	9	
B105; R84	196		7	7	–	–	–	–	–	7	
B142; R96	222		5	–	1	1	–	–	–	2	
Total	945	6.90%	64	26	8	6	1	0	0	41	4.30%
**Middle frequency**											
B79; R63	217		13	8	1	1	–	–	–	10	
B82; R98	187		8	6	1	–	–	–	–	7	
B103; R83	154		18	10	2	–	1	–	–	13	
B106; R84	159		11	8	–	1	–	–	–	9	
B141; R96	133		15	5	2	2	–	–	–	9	
Total	850	7.30%	65	37	6	4	1	0	0	48	5.60%
**Low frequency**											
B80; R63	140		10	8	1	–	–	–	–	9	
B86; R99	168		17	7	1	3	–	–	–	11	
B104; R83	174		7	3	2	–	–	–	–	5	
B107; R84	203		12	8	–	–	1		–	9	
B143; R96	210		4	4	–	–	–	–	–	4	
Total	895	9.30%	50	30	4	3	1	0	0	38	4.20%
**24 months**											
**High frequency**											
B111; R101	199		13	10	–	1	–	–	–	11	
B114; R102	162		5	3	1	–	–	–	–	4	
B117; R100	129		7	6	1	–	–	–	–	7	
B135; R109	170		8	5		1	–	–	–	6	
B138; R110	172		35	8	4	5	1	–	–	18	
Total	832	18%	68	32	6	7	1	0	0	46	5.50%
**Middle frequency**											
B112; R101	124		16	5	4	2				11	
B115; R102	187		24	8	4	1		1		14	
B118; R100	144		19	9	2	2				13	
B121; R108	127		11	5	3					8	
B136; R109	142		16	10	1		1			12	
Total	724	21%	86	37	14	5	1	1	0	58	8.01%
**Low frequency**											
B113; R101	170		16	11	1	1	–	–	–	13	
B116; R102	139		15	9	3	–	–	–	–	12	
B119; R100	155		16	4	3	2	–	–	–	9	
B122; R108	181		15	5	5	–	–	–	–	10	
B137; R109	156		24	12	4	–	1	–	–	17	
Total	801	18.90%	86	41	16	3	1	0	0	61	7.60%
**28–29 months**											
**High frequency**											
B87; R29	135		8	6	1	–	–	–	–	7	
B90; R46	172		18	7	4	1	–	–	–	12	
B93; R79	136		9	6	–	1	–	–	–	7	
B99; R45	159		15	8	2	1	–	–	–	11	
B126; R47	165		26	11	2	1	2	–	–	16	
Total	767	24.40%	76	38	9	4	2	0	0	53	6.90%
**Middle frequency**											
B88; R99	138		5	5	–	–	–	–	–	5	
B91; R46	130		22	7	2	2	–	1	–	12	
B94; R79	129		11	7	–	–	1	–	–	8	
B100; R45	130		22	13	1	1	1	–	–	16	
B127; R47	153		29	8	6	3	–	–	1 (6)	18	
Total	680	25.80%	89	40	9	6	2	1	1	59	8.70%
**Low frequency**											
B89; R99	168		9	3	3	–	–	–	–	6	
B92; R46	142		7	4	–	1	–	–	–	5	
B95; R79	144		3	3	–	–	–	–	–	3	
B101; R45	159		7	7	–	–	–	–	–	7	
B128; R47	182		27	4	8	1	1	–	–	14	
Total	795	19.50%	53	21	11	2	1	0	0	35	4.40%

“BXX” designates the block number. “RXX” designates the animal.

### 2.2 Perfusion and sectioning

Each animal was deeply anesthetized with isoflurane and perfused transcardially with Tyrode’s solution, followed by 250 ml of 2% glutaraldehyde and 2% paraformaldehyde [with one exception; case R79, Blocks 93,94, and 95 (Tables 1, 2) was perfused and stored with 3% glutaraldehyde and 1% paraformaldehyde] in 0.1 M phosphate buffer at a pH 7.4. The brain was removed and stored at 4°C in 2% glutaraldehyde and 2% paraformaldehyde in 0.1 M phosphate buffer. The following day the brain was prepared for processing by removing the cerebellum and cortex and blocking the remaining piece with transverse cuts posterior to the cochlear nucleus and anterior to the thalamus. The tissue was then cut into 50 μm thick transverse section with a Vibratome (VT1000S, Leica Microsystems, Buffalo Grove, IL, USA). The tissue was collected in six series. Series were processed as described below or stored in freezing buffer for future processing.

### 2.3 Tissue processing for EM

**FIGURE 1:**
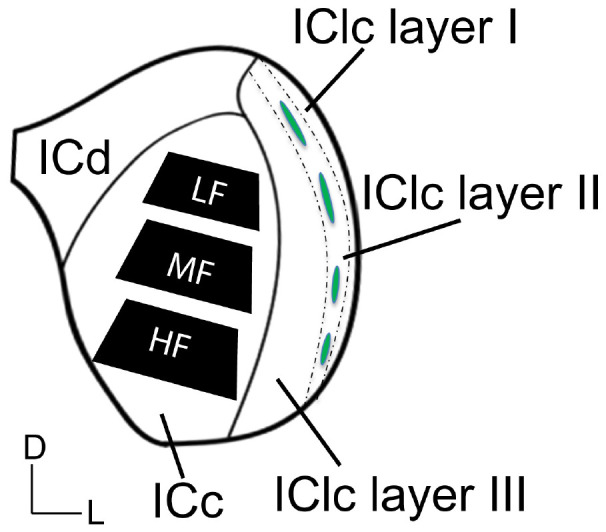
Schematic illustrating three subdivisions of the inferior colliculus in the coronal plane. The black trapezoids indicate the regions of ICc across the ventromedial-dorsolateral axis where tissue was extracted from in each case. Dashed lines demonstrate the approximate borders between the three layers of the lateral cortex of the inferior colliculus (IClc). Green ovals indicate the approximate locations of the GABAergic modules that are often found and define anatomical features of the second IClc layer. D, dorsal; L, lateral; HF, high frequency region; ICc, central inferior colliculus; ICd, dorsal cortex of the IC; IClc layer I, the first layer of the lateral cortex of the IC; IClc layer II, the second layer of the IClc; IClc layer III, the third layer of the IClc; LF, low frequency region; MF, middle frequency region.

A series of tissue was post-fixed in 1% osmium tetroxide for 30 min, dehydrated in a series of alcohols (50%, 70%, 95%, 100% and 2× propylene oxide; each run was 10 min), embedded in Durcupan resin (Sigma-Aldrich; Millipore Sigma, Burlington, MA, USA) and flat-mounted between sheets of Aclar Embedding Film (Ted Pella, Inc., Redding, CA, USA) at 60°C for 48–72 h. Mid-rostrocaudal IC sections (between interaural levels 0.24 and 0.36 mm; Paxinos and Watson, 1998) were examined with brightfield stereomicroscopy. Trapezoidal blocks, with a 0.75 mm base and 0.5–0.6 mm height, were extracted across the ventromedial-dorsolateral axis of the ICc (Figure 1). Three “blocks” of tissue were taken from each animal processed in the study. The ventromedial-dorsomedial axis of the rat ICc, after fixation, is approximately 2 mm. To better avoid our dorsolateral-most block including lateral or dorsal cortex of the IC, the total length of tissue taken across the axis was ∼1.8 mm (0.6 mm height per block). Initial borders of the ICc were delineated according to the rat anatomical atlas of the brain (Paxinos and Watson, 1998). Osmium fixation revealing the conspicuous lateral lemniscal fibers, libraries of decarboxylase (GAD) immunoreactivity in EM prepared tissue, adjacent sections reacted for Nissl, and our experience with EM in the IC further guided our block trimming to best ensure tissue was from ICc (Nakamoto et al., 2013; Mellott et al., 2014; Mafi et al., 2022). More specific details on the trimming process can be found in Mafi et al. (2022). Tissue blocks were glued to a cylindric resin base with cyanoacrylate (Krazy Glue, Columbus, OH, USA). IC sections with removed tissue blocks were then imaged for record keeping and representative comparison between cases. We refer to the ventromedial-most ICc block as representing the high frequency region, the middle-most block representing the middle frequency region, and dorsolateral-most block representing lower frequencies (Figure 1). We did not record from the ICc; however we adopt this naming convention for the sake of conciseness, brevity, and convention as the tonotopic axis is well established.

Ultrathin sections were taken at a thickness of 50 nm with an ultramicrotome (UC6 Ultramicrotome, Leica Microsystems, Buffalo Grove, IL, USA). For each block of tissue, every twelfth section was collected onto a 200 or 300-mesh Formvar coated nickel mesh grid (Electron Microscopy Science, Hatfield, PA, USA) to ensure a singular synapse was not collected across two grids and analyzed twice. A total of eight grids, each with a single ultrathin section of layer of the ICc, were collected per block of tissue. Briefly, see Nakamoto et al. (2013) and Mellott et al. (2014), sections dried for 3 h and were then placed overnight into anti-GABA antibody (rabbit anti-GABA, Sigma, St. Louis, MO, USA) diluted 1:500 in 0.05 M Tris-buffered saline with 0.1% Triton X-100, pH 7.6 (TBST), washed in TBST pH 7.6, then washed in TBST pH 8.2, and placed into a secondary antibody conjugated to 15 nm gold particles (goat anti-rabbit, diluted 1:25 in TBST pH 8.2; Ted Pella Inc., Redding, CA, USA). Lastly, sections were washed in TBST pH 7.6, washed in Nanopure water, stained with uranyl acetate (2% aqueous) and Reynold’s lead citrate (Reynolds, 1963), and dried.

### 2.4 EM imaging

Sixty blocks of tissue from 20 male FBN rats with superior ultrastructure were chosen for imaging and quantification. We use a 5-point scale to grade the intactness and quality of ultrastructure in each case. Only tissue with a score of 4 or 5 was quantified. Our 5-point scale reflects a combination of successful fixation, immunogold processing and absence of electron dense artifacts. Nine of the 15 cases were scored as a “5” and the remaining six cases were a “4.” Scores of 4 and 5 yield clear ultrastructure with easily identifiable profiles that are readily resolved. The distinction between a 4 and a 5 is commonly due to a fold in the tissue or excess precipitate, which we avoid when imaging. Tissue scored as a 3 yields ultrastructure that can be qualitatively analyzed; however membrane integrity is not preserved such that quantitative data can be consistently extracted. All images presented are from a case scored as a 4/5. Tissue scored as a 1 or 2 has severe defects in the pre- and postsynaptic membranes such that synaptic profiles are difficult to interpret. Ultrastructure of the ICc was imaged with a transmission electron microscope (JEM-1400Plus, JEOL, Peabody, MA, USA) at an accelerating voltage of 80 kV and at a magnification of 50,000. Based on experience, a magnification of 50,000 ensures that all inhibitory synapses in the inferior colliculus are visible. Tissue was digitally imaged and rendered with an Orius 100 keV or Rio9 side mount camera (Gatan, Pleasanton, CA, USA). Images of ultrastructure were taken with Gatan Microscopy Suite Software (GMS3, Gatan, Pleasanton, CA, USA) integrated and calibrated with SerialEM Tomography software (Mastronarde, 2003). SerialEM is a gold standard for analytical applications in biological TEM and allowed us to image and analyze and add data at higher rate of efficiency. For each tissue block, we collected 400 μm^2^ (22,214 × 22,214 pixels) montages across 8 grids for a total of 3,200 μm^2^. All attempts were made such that each montage was collected from the center of each ultrathin section. Montages were analyzed by individuals blind to the age and ICc region imaged. Adobe Photoshop (Adobe Systems, Inc., San Jose, CA, USA) was used to add scale bars, crop images, adjust intensity levels and colorize monochrome images.

### 2.5 Analysis of inhibitory and excitatory profiles

We recorded each DCV that was present in a bouton, axon, and dendrite. Our classification of ultrastructure is largely based on the criteria defined by Peters et al. (1991) and our previous work (Mellott et al., 2014; Nakamoto et al., 2014; Mafi et al., 2022). Briefly, boutons have a “pool” of vesicles with clear centers and generally an absence of a cytoskeleton. We never observed a bouton with a DCV without clear vesicles. Dendrites were identified through a combination of criteria (irregular contours, spines, presence of synaptic inputs, and free ribosomes). Axons were commonly myelinated, and axons do not commonly contain free ribosomes in their cytoplasm. Identifying small unmyelinated axons from small dendrites provides some difficulty (Peters et al., 1991). Generally speaking, small axons in the IC travel as bundles through the neuropil while dendrites travel in irregular patterns and are not typically in bundles. Additionally, the presence of ribosomes can help identify dendrites. When identifying a presynaptic terminal with a DCV we first note if the postsynaptic density is symmetric or asymmetric. We then classified each profile (bouton, axon, and dendrite) as non-GABAergic or GABAergic based on the accumulation of gold particles as compared to background (Nakamoto et al., 2013; Mellott et al., 2014; Mafi et al., 2022). In our previous report on the aging ultrastructure of the IC, we found that immunogold labeling was reduced in the aged tissue (Mafi et al., 2022). To maintain consistency between studies, older structures that were characterized as GABAergic had to have a density of gold particles that was at least three times greater than background. Although the goal of the current study was not synaptic analysis, when a DCV was identified in a bouton forming a synapse, we characterized the synapse as either excitatory or GABAergic for qualitative analysis. Synapses were classified by: (1) vesicle shape [pleomorphic vesicles (inhibitory) or round vesicles (excitatory)] and, (2) postsynaptic densities forming symmetric (pre- and postsynaptic membranes were of similar thickness; inhibitory) or asymmetric (postsynaptic densities were conspicuously thicker than the presynaptic densities; excitatory) junctions (Rockel and Jones, 1973; Paloff and Usunoff, 1992; Helfert et al., 1999; Nakamoto et al., 2013). Detailed descriptions characterizing IC ultrastructure with post-embedding immunogold techniques to label GABAergic profiles can be found in Nakamoto et al. (2013) and Mafi et al. (2022).

### 2.6 Data analysis

We examined 48,000 μm^2^ of ICc across five blocks of 3–4-month-old tissue, 48,000 μm^2^ of ICc across five blocks of 19–20-month-old tissue, 48,000 μm^2^ of ICc across five blocks of 24-month-old tissue, and 48,000 μm^2^ of ICc across five blocks of 28–29-month-old tissue with ImageJ (Schneider et al., 2012; Table 1). As boutons and synapses are known to be reduced in the aging IC, we quantified each bouton in each tissue block of each case to better understand age-related changes to the presence of DCVs in boutons (Helfert et al., 1999; Mafi et al., 2022).

Variation in number of DCVs by ultrastructural location according to age group, was analyzed using one-way analysis of variance (ANOVA) models for each region. Six separate ANOVAs were run with age as the independent variable and number of DCVs as the outcome. Tukey’s Honest Significant Difference (HSD) was run for pairwise *post-hoc* tests of differences between consecutive age groups (i.e., 3–4 months versus 19–20 months, 19–20 months versus 24 months, 24 months versus 28–29 months) and pairwise values were adjusted using the false discovery rate procedure (Benjamini and Hochberg, 1995), a method that simultaneously limits experiment-wise alpha inflation and minimizes the correlated loss of statistical power. All statistical tests were performed in R (version 3.6.3 for Mac OS X; R Core Team, 2020), supplemented by the add-on packages *nlme compareGroups* (Subirana et al., 2014).

## 3 Results

We examined the ultrastructural location of DCVs in non-GABAergic and GABAergic terminals, axons, and dendrites across four age groups (3–4 months, 19–20 months, 24 months, and 28–29 months) in the central inferior colliculus (ICc). We analyzed 3,811 DCVs in excitatory profiles and 1,567 DCVs in inhibitory profiles across 192,000 μm^2^ of tissue (Table 1). DCVs were most commonly located in non-GABAergic profiles at each age (Table 1). The most robust age-related increases of DCVs also occurred in non-GABAergic profiles (Table 1). We first describe DCV ultrastructure across the ventromedial-dorsolateral axis of the ICc. We then present data regarding age-related increases of DCVs in the dendrites of IC cells. Lastly, we qualitatively describe the frequency of DCVs in non-GABAergic and GABAergic boutons in the aging IC.

### 3.1 DCVs can be found across the ICc, regardless of age

**FIGURE 2:**
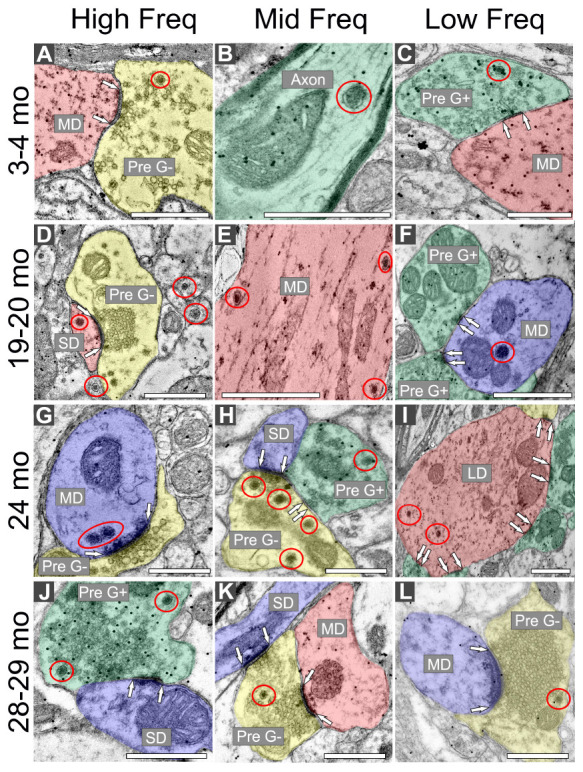
Electron micrographs across four age groups showing dense core vesicle (DCV) ultrastructure across the ventromedial-dorsolateral axis of the central inferior colliculus (ICc). GABAergic presynaptic (Pre G+) terminals are pseudocolored green. GABAergic postsynaptic targets are pseudocolored red. GABA-negative presynaptic (Pre G–) terminals are pseudocolored yellow. GABA-negative postsynaptic targets are pseudocolored blue. Black dots demonstrate immunogold labeling of GABA. Synapses are indicated by pairs of white arrows. **(A–C)** Electron micrographs showing examples of DCVs in 3–4 month old tissue across the ventromedial-dorsolateral axis of the ICc. **(D–F)** Electron micrographs showing examples DCVs in 19–20 month old tissue across the ventromedial-dorsolateral axis of the ICc. **(G–I)** Electron micrographs showing examples DCVs in 24 month old tissue across the ventromedial-dorsolateral axis of the ICc. **(J–L)** Electron micrographs showing examples DCVs in 28 month old tissue across the ventromedial-dorsolateral axis of the ICc. DCVs are circled in red. LD, large dendrite; MD, medium dendrite; SD, small dendrite. Scale bars, 500 nm.

Regardless of age and ventromedial-dorsolateral location, DCVs were found in dendrites, terminals, and axons in the ICc (Figure 2). We observed DCVs in both GABAergic and non-GABAergic presynaptic terminals in the ICc (Figures 2A, C, H, J–L). DCVs were also found in GABAergic and non-GABAergic dendrites (Figures 2D–G, I). Often, DCVs located in dendrites were near the postsynaptic density (Figures 2D, F, G, I). We did not observe DCVs at dendrodendritic synapses; it appears that dendrodendritic synapses in the rat IC are rare. At 28–29 months DCVs were found in presynaptic terminals forming non-prototypical synapses [e.g., GABAergic synapse forming an apparent asymmetric synapse (Figure 2J), non-GABAergic synapse with a subsynaptic body (Figure 2L)]. However, it was rare to observe a DCV bound to the pre- or postsynaptic membrane (Figure 2).

### 3.2 DCVs increase with age in non-GABAergic dendrites in low- and middle-frequency areas of the ICc

**FIGURE 3:**
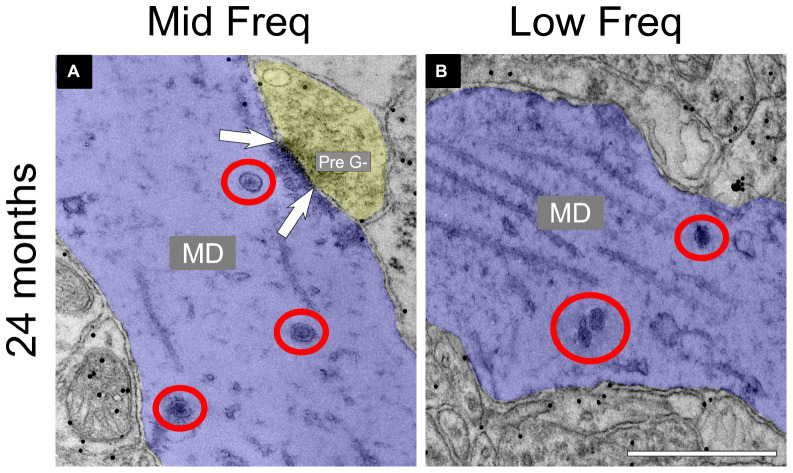
Electron micrographs at 24 months showing dense core vesicle (DCV) ultrastructure in the middle and low frequency regions of the central inferior colliculus (ICc). GABA-negative dendrites are pseudocolored blue. A GABA-negative presynaptic (Pre G–) terminal is pseudocolored yellow. Black dots demonstrate immunogold labeling of GABA. Synapses are indicated by pairs of white arrows. **(A)** Electron micrograph showing a group of DCVs in a non-GABAergic dendrite at 24 months in the middle frequency region of the ICc. **(B)** Electron micrograph showing a group of DCVs in a non-GABAergic dendrite at 24 months in the low frequency region of the ICc. DCVs are circled in red. MD, medium dendrite. Scale bars, 500 nm.

**FIGURE 4:**
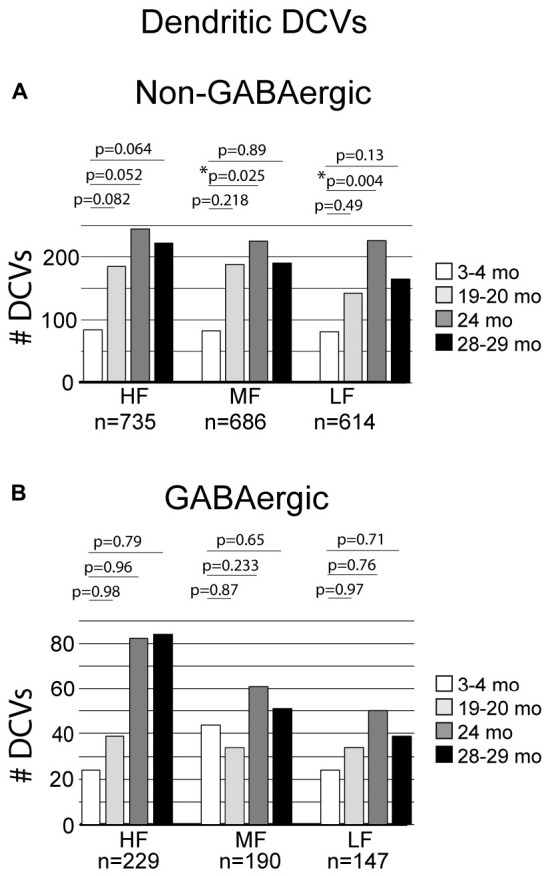
Bar graphs summarizing the number of DCVs found in non-GABAergic and GABAergic dendrites. **(A)** Pairwise differences demonstrated a significant increase of DCVs in non-GABAergic dendrites in the middle frequency region between 3–4 and 24 months (*p = 0.025). Pairwise differences also revealed a significant increase of DCVs in non-GABAergic dendrites in the low frequency region at 24 months (*p = 0.004). **(B)** Pairwise differences demonstrated no significant age-related differences in the number of DCVs from 3 to 4 months across GABAergic dendrites in the high frequency region (19–20 months, *p* = 0.98; 24 months, *p* = 0.96; 28–29 months, *p* = 0.79), middle frequency region (19–20 months, *p* = 0.87; 24 months, *p* = 0.233; 28–29 months, *p* = 0.65), and low frequency region (19–20 months, *p* = 0.97; 24 months, *p* = 0.76; 28–29 months, [*p* = 0.71]). Each age group had five cases.

The total number of DCVs in non-GABAergic dendrites at 3–4 months across the high (ventromedial), middle and low (dorsolateral) frequency regions was 84, 83, and 81, respectively (Table 1). By 19–20 months these values doubled across the IC and increased further at 24 months (Table 1). When DCVs were observed in dendrites, they were typically singular and not near other DCVs. However at older ages in the low and middle frequency regions, while still uncommon, we observed DCVs near each other (Figure 3). In the low frequency region, the number of DCVs significantly increased from 83 to 226 at 24 months (*p = 0.004; Figure 4A). In the middle frequency region, the number of DCVs significantly increased from 83 to 255 at 24 months (*p = 0.025; Figure 4A). However the age-related increase of DCVs was not found to be significant at 19–20 months and 28–29 months (Figure 4A). In the high frequency region, despite an increase in the number of DCVs with age, our models did not detect a significant increase with age (Figure 4A).

### 3.3 DCVs do not significantly increase with age in GABAergic dendrites in the ICc

**FIGURE 5:**
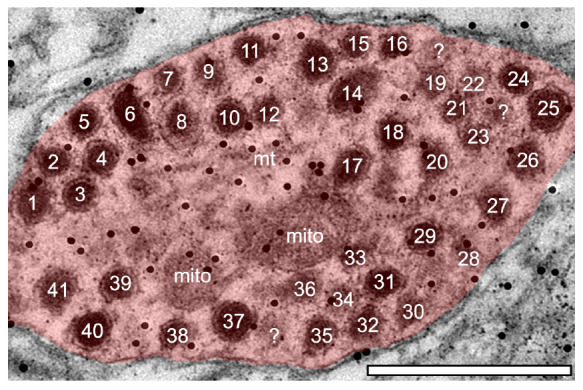
Electron micrograph of a medium sized GABAergic dendrite at 24-months from the ventromedial (high frequency region) of the central IC with over 40 DCVs. Although rare, GABAergic dendrites packed with DCVs could be found in the aged IC. Black dots demonstrate immunogold labeling of GABA. mito, mitochondria; ?, likely DCV. Scale bar, 500 nm.

Although the total raw number of DCVs increased at older ages in the GABAergic dendrites, we found no significant change from 3 to 4 months in the high frequency region, middle frequency region and low frequency region (Figure 4B). A contributing factor to the non-significant findings appears to be the variability of DCV pools in the aging GABAergic dendrites. During aging we observed a few aged GABAergic dendrites in cross-section with dozens of DCVs (Figure 5). Thus, it appears that a few select neurons in the ICc may retrogradely transport neurotrophins and/or neuropeptides at great quantities during aging.

### 3.4 Non-GABAergic boutons with DCVs are more likely to be spared in aging

**FIGURE 6:**
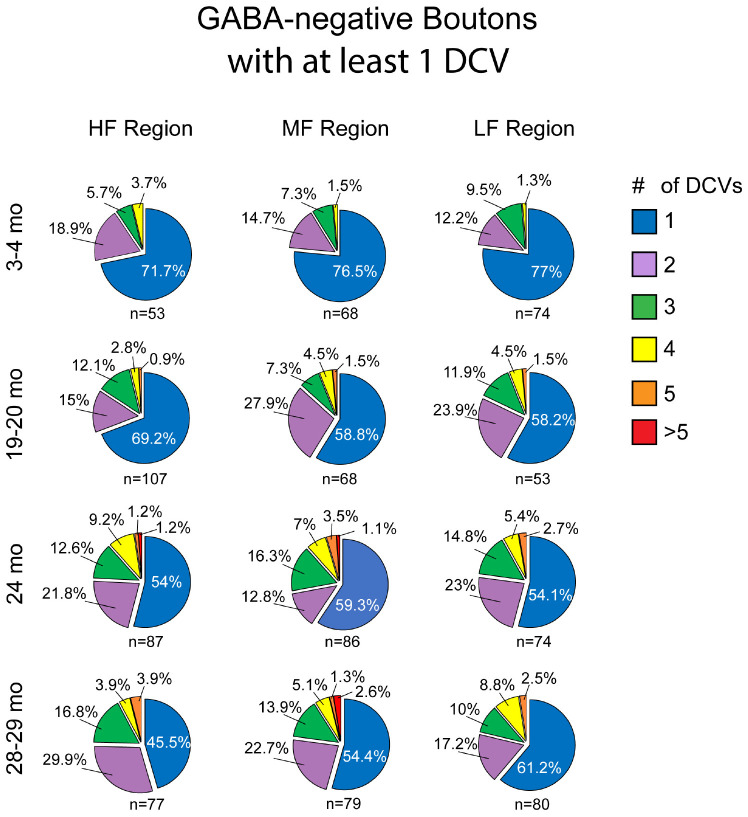
Pie charts showing the distribution of non-GABAergic boutons that had at least one DCV across four age groups from tissue representing the high, middle, and low frequency regions of the ICc. Regardless of ICc region, when a bouton had a DCV at 3–4 months, having just one was the most common (blue). We did not observe a 3–4 month non-GABAergic bouton with five or more. At the three later ages it became more common for non-GABAergic boutons that had a DCV to have three (green), four (yellow), five (orange), or more (red). The *n*’s represent the number of boutons that had at least one DCV. HF, high frequency; MF, middle frequency; LF, low frequency.

**FIGURE 7:**
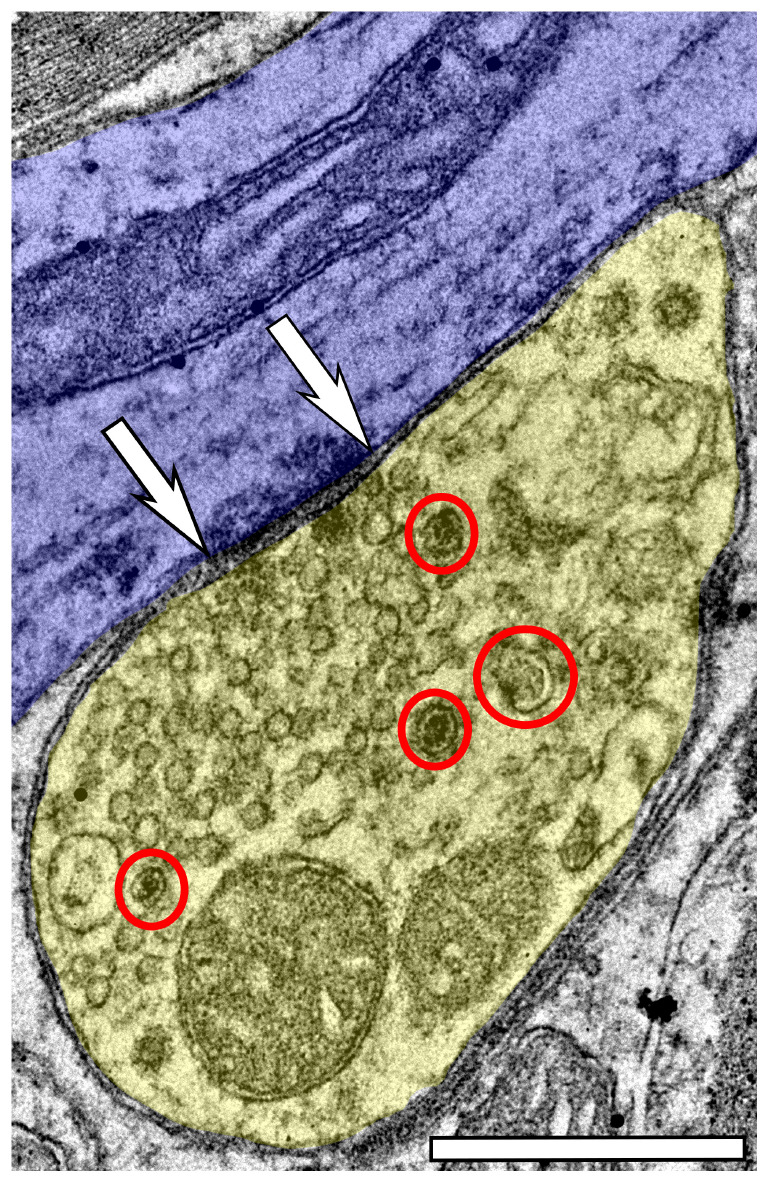
Electron micrograph of a non-GABAergic terminal from the dorsolateral (low frequency region) of the ICc at 28–29 months with at least four DCVs forming an asymmetric synapse onto a non-GABAergic medium dendrite. DCVs are circled in red. Scale bar, 500 nm.

Approximately 6% of non-GABAergic boutons had one or more DCVs at 3–4 months of age (Table 2). The majority of these boutons had just one DCV, regardless of location in the ICc (Figure 6 and Table 2). At later ages, it became more common to find non-GABAergic boutons with at least one DCV, and boutons with multiple DCVs also increased (Figures 6, 7 and Table 2). These boutons with multiple DCVs commonly made synapses onto non-GABAergic dendrites (Figure 7). The most conspicuous increase of DCVs occurred in the high frequency ICc at 19–20 months, and the high and middle frequency regions at 24 months (Figure 6 and Table 2). At 3–4 months of age, the low frequency ICc had the greatest raw number (100) of DCVs (Table 2). However, by 19–20 months the number of DCVs in the high frequency/ventromedial region had more than doubled (75–162), and the percentage of non-GABAergic boutons with at least one DCV increased from 5.24% to 11.2% (Table 2). At 24 months, the number of DCVs in the middle frequency region of the ICc doubled (68–160), and the percentage of boutons with at least one DCV increased from 6.6% to 10.5% (Table 2). Increases in the number of DCVs in the low frequency region were less robust between 3–4 months and 24/28–29 months (100–133), and the percentage of boutons having at least one DCV between 3–4 months and 28 months increased slightly (6.8%–8.7%; Table 2). Overall, these increases were largely driven by an increasing population of non-GABAergic boutons having four or more DCVs (Figure 7 and Table 2).

As boutons and synapses are known to be downregulated in the aging IC, we quantified each non-GABAergic bouton in our experiments to better understand what proportion of boutons contain DCVs during aging. At 3–4 months we found a consistent number of boutons across the ventromedial-dorsolateral axis of the ICc (high-1,067; middle-1,031; low-1,096: Table 2). In the high frequency region at 19–20 month, 24 months, and 28–29 months there was a 9.1%, 23.8%, and 29.7% reduction of non-GABAergic boutons, respectively (Table 2). In the middle frequency region at 19–20 months, 24 months, and 28–29 months there was a 7.4%, 20.9%, and 24.8% reduction of non-GABAergic boutons, respectively (Table 2). Lastly, in the low frequency region at 19–20 months, 24 months, and 28–29 months there was a 8.0%, 15.8%, and 17.6% reduction of non-GABAergic boutons, respectively (Table 2). Taken together with the data mentioned above, our findings suggest that non-GABAergic cells/boutons that package DCVs are more likely to be spared during aging than non-GABAergic cells/boutons that do not package DCVs.

### 3.5 GABAergic boutons with DCVs are more likely to be spared in aging

**FIGURE 8:**
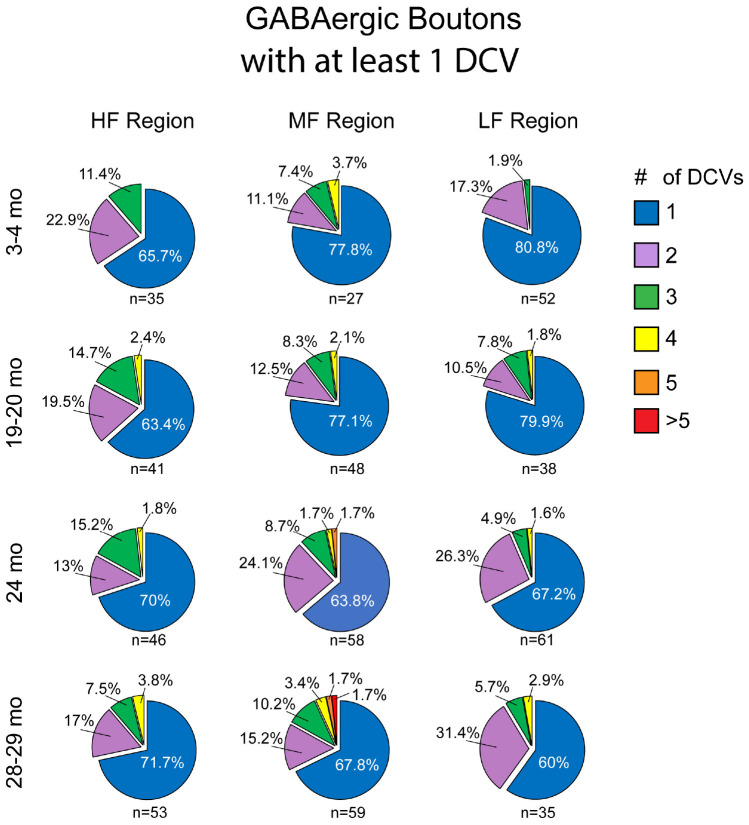
Pie charts showing the distribution of GABAergic boutons that had at least one DCV across four age groups from tissue representing the high, middle, and low frequency regions of the ICc. Regardless of ICc region, when a bouton had a DCV at 3–4 months, having just one was the most common (blue). We did not observe a 3–4 month GABAergic bouton with five or more. At the three later ages there was a slight increase in the number of GABAergic boutons with four (yellow) or more (orange and red) DCVs. The *n*’s represent the number of boutons that had at least one DCV. HF, high frequency; MF, middle frequency; LF, low frequency.

Similar to non-GABAergic boutons, at any age and/or ICc location GABAergic boutons that contained DCVs typically had just one (Figure 8). GABAergic boutons with multiple DCVs occurred more routinely during aging in the middle and low frequency regions (Figure 8). However, at older ages, the proportion of GABAergic boutons with just one DCV increased in the high frequency ICc (Figure 8). As with the non-GABAergic population, during aging there was a greater raw number of DCVs in GABAergic boutons and a higher number of GABAergic boutons with at least one DCV in the high and middle frequency regions of the ICc (Figure 8 and Table 3). The raw number of DCVs in GABAergic boutons and number of GABAergic boutons with at least one DCV peaked at 24 months, but was lowest at 28 months (Figure 8 and Table 3).

We also quantified each GABAergic bouton in our experiments. Unsurprisingly, there was an overall reduction of GABAergic boutons at each age and at each ICc region (Table 3). The percentage of GABAergic boutons across the ICc with a DCV was only ∼4% at 3–4 months and increased during aging, with greater variability in the low frequency ICc (Table 3). Taken together we come to the same conclusion as we have with the non-GABAergic population: GABAergic cells/boutons that package DCVs are more likely to be spared during aging than GABAergic cells/boutons that do not package DCVs.

## 4 Discussion

The current study describes populations of DCVs in the aging ICc. Our findings demonstrate that DCVs are infrequent in the young rat ICc. This agrees with studies conducted in cat (Paloff and Usunoff, 1992). However, our data demonstrate that DCVs increase with aging in the rat ICc. Specifically we found the greatest increase of DCVs at 24 months in non-GABAergic dendrites throughout the middle and dorsolateral portions of the ICc’s ventromedial-dorsolateral tonotopic axis (corresponding to mid- and low-frequency representation). This is interesting as the FBN rat has been shown to lose low frequency hearing around 24 months of age (Keithley et al., 1992; Caspary et al., 2005; Cai et al., 2018). While the raw number of DCVs in boutons did not significantly change during aging, there was (1) an increase in the number of boutons with multiple DCVs, and (2) the percentage of all boutons that had at least one DCV increased with age. Given that we broadly found a ∼25% loss of boutons across the ICc, which reflects findings by Helfert et al. (1999), we interpret our data to imply that presynaptic boutons in the ICc which are lost with age are not likely releasing contents that would be packaged by a DCV. As this is the first report of age-related changes to DCVs in the IC, future studies will hopefully determine what neuropeptides, neurotrophins and/or presynaptic proteins are undergoing age-related changes in the IC.

### 4.1 Technical considerations

The FBN rat is a recommended aging model by the National Institute on Aging as it has a longer median lifespan than other strains of mice and rats (Lipman et al., 1996; Lipman, 1997). Of note, the FBN rat is routinely used as a characterized model for aging, in particular for studies of the central auditory system (Caspary et al., 2008; Cai et al., 2018; Caspary and Llano, 2018; Robinson et al., 2019; Mafi et al., 2020, 2021, 2022; Kommajosyula et al., 2021). We have chosen to use four age groups in this study. Our 3–4 month and 28–29 month groups are standard ages for “young” and “old,” when there are no hearing deficits and very well characterized hearing loss, respectively. The use of two middle ages, a 19–20 month group and 24 month group, reflect ages when hearing deficits are not commonly reported and when hearing thresholds are significantly elevated, respectively (Cai et al., 2018). However, we acknowledge that the hearing thresholds of the FBN rats in the current study were not measured and we do not know if a 19 month old rat had perfect hearing or if a 24 month old rat had poor hearing.

In the current study we equate parts of the ICc with tonotopic regions (e.g., low-frequency region). We did not characterize IC cells with electrophysiological techniques in the current study. We took the ventromedial-dorsomedial axis of the ICc tissue and divided it into thirds. While we do not know the characteristic frequencies represented in each tissue block, as the tonotopic axis of the IC is well established, we have no reason to believe that our ventromedial most block does not represent higher frequencies compared to the other two blocks; and the dorsolateral most block would represent the lowest frequencies. Another consideration in the interpretation of our data is that, although a common view of the IC’s tonotopic map is one of a continuous gradient, the rat IC tonotopic map may be organized in a stepwise manner (Malmierca et al., 2008). Thus, our middle-most ICc blocks may represent frequencies closer to one end of the tonotopic map than the other.

We only immunolabeled for GABA, thus we cannot know the neurochemical profile of boutons that were not GABAergic. In terms of non-GABAergic boutons that formed symmetric synapses, we presume these are in part glycinergic. That said, the IC receives input from many neuromodulators, and the morphology of their postsynaptic densities are not well classified (Schofield and Hurley, 2018). We hope that this study will serve as a foundation to examine the ultrastructure of neuromodulators and neurotransmitters that are packaged by DCVs in the aging IC.

### 4.2 DCVs in the inferior colliculus

The first ultrastructural description of DCVs in the IC was in cat (Jones and Rockel, 1973; Rockel and Jones, 1973; Paloff et al., 1989). Decades later, boutons containing DCVs were characterized in greater detail and defined a unique type of ICc bouton (Paloff and Usunoff, 1992). The current study broadly agrees with the findings from these original studies: (1) DCVs occurred in boutons with pools of smaller clear vesicles, (2) DCVs were not clustered toward the synapse or membrane, (3) boutons with DCVs commonly had 0–1 mitochondria, (4) DCVs are not uniformly found throughout the ICc, and (5) DCVs are generally uncommon in the ICc (Paloff and Usunoff, 1992). Our findings are further in line with Paloff and Usunoff (1992) in that we found presynaptic terminals with a DCV(s) terminating on a soma to be exceedingly rare (observed only once in our analysis of 192,000 μm^2^ of tissue). However, Rockel and Jones (1973) found that boutons carrying DCVs more routinely terminate on cell bodies.

The current study (in rat) differs from the previous studies (in cat) in two ways. First, work in the cat has demonstrated that when DCVs are present in presynaptic boutons, there may be over one to two dozen (Rockel and Jones, 1973; Paloff and Usunoff, 1992). The current study in rats, and mice (unpublished observations), rarely revealed more than three DCVs in a single bouton. Previous reports of the rat IC ultrastructure also imply that DCVs, let alone pools of them, are rare (Ribak and Roberts, 1986; Roberts and Ribak, 1987). Even though Roberts and Ribak revealed and described the rat IC ultrastructure in considerable detail, only one DCV is visible throughout their images (Ribak and Roberts, 1986; Roberts and Ribak, 1987). Our data also appears to be in agreement with prior studies in rat hippocampus that quantified DCVs, as presynaptic terminals containing DCVs commonly had only a few (Sorra et al., 2006; Tao et al., 2018). Taken together, we believe that the pool size of DCVs in presynaptic terminals is likely species dependent.

Second, we found that the number of DCVs found in dendrites was nearly equal to the number of DCVs found in axons/boutons at young age (Table 1). Furthermore, specifically during middle-age in the dorsolateral half of the ICc, there was a significant age-related increase of DCVs in the ICc dendrites. Previous reports focus on DCVs pools in the IC do not describe a consistent population of DCVs throughout the dendrites of the ICc (Rockel and Jones, 1973; Paloff and Usunoff, 1992). It is unknown if these reports simply did not analyze dendrites for DCVs or if there is a species difference between cats and the current studies that largely utilize rodent and cell culture. However, in the mammalian central nervous system, DCVs pools can be found equally distributed between the dendrites and axons/terminals of a given cell (Persoon et al., 2018). However, DCVs in the axons and boutons are often focused on as their release probability is much higher than the ones in the dendrites (Persoon, et al., 2018). It is important to note DCV fusion events in dendrites are not as tightly time locked to a cell firing and require sustained Ca^2+^ (Xia et al., 2009). Fusion mechanism aside, dendritic exocytosis is a critical function for retrograde signaling, synapse growth and plasticity, and cellular morphology and DCVs play a prominent role in the dendritic release of many neuropeptides and neurotrophins (Regehr et al., 2009; Kennedy and Ehlers, 2011; Ramamoorthy et al., 2011).

A major finding of the current study was that while there was a loss (∼25%) of boutons at old age, the percentage of boutons with DCVs did not change with age. Similar to the dendritic population, the more robust increases occurred in the GABA-negative boutons. We found that non-GABAergic and GABAergic boutons with DCVs made synapses onto both GABAergic and excitatory dendrites (we presume non-GABAergic dendrites in the IC are excitatory as only GABAergic and glutamatergic cells have been demonstrated in the IC, Merchán et al., 2005; Oliver, 2005) at any age. Thus, the contents of DCVs in the aging IC are likely affecting both inhibitory and excitatory circuits.

### 4.3 Functional implications

Broadly, DCVs are divided into small (∼40–80 nm) and large (∼90–200 nm) groups (Peters et al., 1991; Paloff and Usunoff, 1992; Edwards, 1998; Sorra et al., 2006; Trueta and De-Miguel, 2012; Merighi, 2018; Tao et al., 2018). Smaller DCVs tend to package biogenic amines, neuromodulators, and presynaptic machinery, while larger DCVs tend to package neuropeptides, hormones and neurotrophic factors (Peters et al., 1991; Michael et al., 2006; Sorra et al., 2006; Merighi, 2018). The exact diameter that defines a small versus a large DCV appears to be a sliding scale based on location (peripheral or central nervous system) and species, and what ultimately determines the size of a DCV is not well known (Merighi, 2018; Lin et al., 2022). Regardless of diameter, there appears to be considerable differences in the locations, releasing mechanisms, stimulation rates for release, and packaged content of DCVs between the peripheral and central nervous systems, nuclei within in the central nervous system, and between cultured and *in vivo* studies (Hökfelt, 2010; Kennedy and Ehlers, 2011; Persoon et al., 2018). In the central nervous system, much of our knowledge regarding DCVs is obtained from the hippocampus, hypothalamus, and neuromodulatory nuclei (e.g. Raphe and Locus Coeruleus: Sorra et al., 2006; Trueta and De-Miguel, 2012). As this is the first report on the aging DCV population in the IC, further studies are needed to reveal the functional relevance of our data. That said, we would like to comment on a few functions that an age-related increase of DCVs may reflect.

#### 4.3.1 Hearing loss and neuromodulation

GABAergic inhibition is reduced in the aging IC (Caspary et al., 2008; Syka, 2020). This downregulation of GABA may be a compensatory homeostatic response to the loss of peripheral excitation (Caspary et al., 2008; Richardson et al., 2012; Caspary and Llano, 2018). Later in life the ongoing decline in GABAergic neurotransmission likely contributes to disrupted temporal precision and increases in central gain that underlie conditions such as presbycusis, tinnitus, and hyperacusis (Palombi and Caspary, 1996; Frisina, 2001; Brozoski et al., 2002; Walton et al., 2002; Norena, 2011; Parthasarathy and Bartlett, 2011; Auerbach et al., 2019).

Although a number of neuromodulators are released in the IC (e.g., serotonin, dopamine, acetylcholine, and noradrenaline), their roles in the aging IC across middle and old ages have not been thoroughly explored (Klepper and Herbert, 1991; Paloff and Usunoff, 2000; Hurley and Pollak, 2005a; Motts and Schofield, 2009; Hurley and Sullivan, 2012; Schofield and Hurley, 2018; Noftz et al., 2020, 2021). In the context of the current study, serotonin neurotransmission may be a promising avenue to pursue in future studies. First, serotonin is known to be packaged in DCVs in the central nervous system (Bruns et al., 2000; Trueta and De-Miguel, 2012; Kim et al., 2021). Second, serotonin has a myriad of functions in the IC (Hurley and Pollak, 1999, 2001; 2005b; Hurley et al., 2002). Third, it is known that aging and hearing loss affects serotonergic neurotransmission (Tadros et al., 2007; Hall et al., 2012; Shim et al., 2012). It is notable that in each of these studies, serotonin and its receptors increased in the aging IC and during hearing loss. Perhaps the increase of DCVs is correlated with increases of serotonin neurotransmission in the aging IC.

Recent studies in the IC have identified a novel class of GABAergic cells that express the neuropeptide/neuromodulator NPY (Silveira et al., 2020, 2023; Anair et al., 2022). Generally speaking, NPY in the brain provides neuroprotective effects during healthy and pathological aging (Chen et al., 2019; Pain et al., 2022). Interestingly, NPY can be trafficked down dendrites, released postsynaptically, and act on presynaptic and postsynaptic receptors (Ramamoorthy et al., 2011; Yi et al., 2018). Future studies will use immunoEM to determine if NPY is also trafficked by DCVs in the dendrites of GABAergic IC cells. Given that NPY significantly regulates local excitation in the IC, it will be critical to determine how the balance of excitation and inhibition is affected by NPY neurotransmission in the aging IC. Our data show increased DCVs in middle and low frequency IC in a model of presbycusis, so it will be interesting to determine whether serotonin or NPY effects change across the tonotopic axis.

#### 4.3.2 Brain-derived growth factor and plasticity

Another potential peptide contained in IC DCVs is BDNF, a neurotrophin released by DCVs throughout the brain to regulate functions such as synaptic transmission, plasticity, neurite growth, and gene regulation (Kennedy and Ehlers, 2011; Dieni et al., 2012). In the auditory system, BDNF is critical for development and normal acoustic function (Wissel et al., 2006; Singer et al., 2014; Chumak et al., 2016). In the IC, BDNF has been tied to development, acoustic trauma, and dendritic integrity (Hafidi et al., 1996; Sato et al., 2001; Sharma et al., 2009; Meltser and Canlon, 2010). Further studies will need to determine if BDNF DCVs are trafficked to IC axons, dendrites or both as the cellular location of BDNF DCVs differ across reports (Dean et al., 2009; Matsuda et al., 2009; Kennedy and Ehlers, 2011; Dieni et al., 2012; Persoon et al., 2018).

Fibroblast growth factors are another likely protein packaged by IC DCVs. Compellingly, levels of fibroblast growth factor receptor 2 (FGF-2) spike during middle age when hearing deficits are not common, and while there is a decrease at old age, FGF-2 levels are still elevated compared to young brains (Sato et al., 2001). This pattern is very similar to trends in the current study, where DCVs in excitatory dendrites maximally increase at middle age and then decline into old age, yet are still more numerous than at young age. Given that (1) FGF-2 contributes to dendritic arborization and synaptic plasticity, (2) DCVs often carry contents such growth factors that play a role in synaptogenesis, and (3) the aged IC has a loss of dendrites and synapses, the IC may undergo a number of plastic events during middle age (Helfert et al., 1999; Sorra et al., 2006; Li et al., 2020). Further supporting the theory of increased plasticity with aging in the IC, perineuronal nets (organized extracellular matrix that have substantial roles in neural plasticity) increase with aging in the IC (Fader et al., 2016; Bosiacki et al., 2019; Mafi et al., 2020, 2021; Almassri et al., 2023). Taken altogether, the increase of DCVs in the aging IC may contribute to elements of dendritic and synaptic plasticity.

## Data availability statement

The original contributions presented in this study are included in the article/supplementary material, further inquiries can be directed to the corresponding author.

## Ethics statement

The animal study was approved by the Northeast Ohio Medical University Institutional Animal Care and Use Committee. The study was conducted in accordance with the local legislation and institutional requirements.

## Author contributions

JM: Conceptualization, Data curation, Funding acquisition, Investigation, Methodology, Project administration, Resources, Supervision, Validation, Visualization, Writing – original draft, Writing – review & editing. SD: Data curation, Formal analysis, Investigation, Writing – review & editing. JB: Data curation, Formal analysis, Investigation, Writing – review & editing. LA: Writing – original draft, Writing – review & editing. AW: Data curation, Formal analysis, Investigation, Supervision, Writing – review & editing. MI: Formal analysis, Investigation, Writing – review & editing. AO: Investigation, Methodology, Supervision, Writing – review & editing. ES: Data curation, Formal analysis, Investigation, Writing – review & editing. AB: Data curation, Investigation, Writing – review & editing. DA: Data curation, Investigation, Writing – review & editing. BV: Data curation, Investigation, Writing – review & editing. AM: Data curation, Formal analysis, Investigation, Methodology, Writing – review & editing. MB: Data curation, Formal analysis, Investigation, Methodology, Software, Writing – review & editing. NT: Investigation, Project administration, Supervision, Writing – review & editing. JY: Data curation, Formal analysis, Software, Supervision, Writing – review & editing.

## References

[B1] Almassri L. S. Ohl A. P. Iafrate M. C. Wade A. D. Tokar N. J. Mafi A. M. (2023). Age-related upregulation of perineuronal nets on inferior collicular cells that project to the cochlear nucleus. *Front. Aging Neurosci.* 15:1271008. 10.3389/fnagi.2023.1271008 38053844 PMC10694216

[B2] Anair J. D. Silveira M. A. Mirjalili P. Beebe N. L. Schofield B. R. Roberts M. T. (2022). Inhibitory NPY neurons provide a large and heterotopic commissural projection in the inferior colliculus. *Front. Neural Circ.* 16:871924. 10.3389/fncir.2022.871924 35693026 PMC9178209

[B3] Auerbach B. D. Radziwon K. Salvi R. (2019). Testing the central gain model: Loudness growth correlates with central auditory gain enhancement in a rodent model of hyperacusis. *Neuroscience* 407 93–107. 10.1016/j.neuroscience.2018.09.036 30292765 PMC8792806

[B4] Auerbach B. D. Rodrigues P. V. Salvi R. J. (2014). Central gain control in tinnitus and hyperacusis. *Front. Neurol.* 5:206. 10.3389/fneur.2014.00206 25386157 PMC4208401

[B5] Benjamini Y. Hochberg Y. (1995). Controlling the false discovery rate: A practical and powerful approach to multiple testing. *J. R. Stat. Soc. Ser. B* 57 289–300.

[B6] Bosiacki M. Gąssowska-Dobrowolska M. Kojder K. Fabiańska M. Jeżewski D. Gutowska I. (2019). Perineuronal nets and their role in synaptic homeostasis. *Int. J. Mol. Sci.* 20:4108. 10.3390/ijms20174108 31443560 PMC6747153

[B7] Brozoski T. J. Bauer C. A. Caspary D. M. (2002). Elevated fusiform cell activity in the dorsal cochlear nucleus of chinchillas with psychophysical evidence of tinnitus. *J. Neurosci.* 22 2383–2390. 11896177 10.1523/JNEUROSCI.22-06-02383.2002 PMC6758251

[B8] Bruns D. Riedel D. Klingauf J. Jahn R. (2000). Quantal release of serotonin. *Neuron* 28 205–220. 10.1016/s0896-6273(00)00097-0 11086995

[B9] Cai R. Montgomery S. C. Graves K. A. Caspary D. M. Cox B. C. (2018). The FBN rat model of aging: Investigation of ABR waveforms and ribbon synapse changes. *Neurobiol. Aging* 62 53–63. 29107847 10.1016/j.neurobiolaging.2017.09.034 PMC5743589

[B10] Caspary D. M. Ling L. Turner J. G. Hughes L. F. (2008). Inhibitory neurotransmission, plasticity and aging in the mammalian central auditory system. *J. Exp. Biol.* 211 1781–1791. 18490394 10.1242/jeb.013581 PMC2409121

[B11] Caspary D. M. Llano D. A. (2018). “Aging process in the subcortical auditory system,” in *The Oxford Handbook of the Auditory Brainstem*, ed. Kandler K. (Oxford: Oxford Press), 1–45. 10.1093/oxfordhb/9780190849061.013.16

[B12] Caspary D. M. Schatteman T. A. Hughes L. F. (2005). Age-related changes in the inhibitory response properties of dorsal cochlear nucleus output neurons: Role of inhibitory inputs. *J. Neurosci.* 25 10952–10959. 16306408 10.1523/JNEUROSCI.2451-05.2005 PMC6725883

[B13] Chen X. Du Y. Chen L. (2019). Neuropeptides exert neuroprotective effects in Alzheimer’s Disease. *Front. Mol. Neurosci.* 11:493. 10.3389/fnmol.2018.00493 30687008 PMC6336706

[B14] Chumak T. Rüttiger L. Lee S. C. Campanelli D. Zuccotti A. Singer W. (2016). BDNF in lower brain parts modifies auditory fiber activity to gain fidelity but increases the risk for generation of central noise after injury. *Mol. Neurobiol.* 53 5607–5627. 10.1007/s12035-015-9474-x 26476841 PMC5012152

[B15] Dean C. Liu H. Dunning F. Chang P. Jackson M. Chapman E. (2009). Synaptotagmin-IV modulates synaptic function and long-term potentiation by regulating BDNF release. *Nat. Neurosci.* 12 767–776. 19448629 10.1038/nn.2315 PMC2846764

[B16] Dieni S. Matsumoto T. Dekkers M. Rauskolb S. Ionescu M. S. Deogracias R. (2012). BDNF and its pro-peptide are stored in presynaptic dense core vesicles in brain neurons. *J. Cell Biol.* 196 775–788. 10.1083/jcb.201201038 22412021 PMC3308691

[B17] Edwards R. H. (1998). Neurotransmitter release: Variations on a theme. *Curr. Biol.* 8 R883–R885. 10.1016/s0960-9822(07)00551-9 9843673

[B18] Fader S. M. Imaizumi K. Yanagawa Y. Lee C. C. (2016). Wisteria floribunda agglutinin-labeled perineuronal nets in the mouse inferior colliculus, thalamic reticular nucleus and auditory cortex. *Brain Sci* 6:13. 10.3390/brainsci6020013 27089371 PMC4931490

[B19] Frisina R. D. (2001). Subcortical neural coding mechanisms for auditory temporal processing. *Hear. Res.* 158 1–27. 11506933 10.1016/s0378-5955(01)00296-9

[B20] Goman A. M. Lin F. R. (2016). Prevalence of hearing loss by severity in the United States. *Am. J. Public Health* 106 1820–1822. 10.2105/AJPH.2016.303299 27552261 PMC5024365

[B21] Goyer D. Silveira M. A. George A. P. Beebe N. L. Edelbrock R. M. Malinski P. T. (2019). A novel class of inferior colliculus principal neurons labeled in vasoactive intestinal peptide-Cre mice. *eLife* 8:e43770. 10.7554/eLife.43770 30998185 PMC6516826

[B22] Hafidi A. Moore T. Sanes D. H. (1996). Regional distribution of neurotrophin receptors in the developing auditory brainstem. *J. Comp. Neurol.* 367 454–464. 8698904 10.1002/(SICI)1096-9861(19960408)367:3<454::AID-CNE10>3.0.CO;2-H

[B23] Hall I. C. Sell G. L. Chester E. M. Hurley L. M. (2012). Stress-evoked increases in serotonin in the auditory midbrain do not directly result from elevations in serum corticosterone. *Behav. Brain Res.* 226 41–49. 10.1016/j.bbr.2011.08.042 21907246

[B24] Helfert R. H. Sommer T. J. Meeks J. Hofstetter P. Hughes L. F. (1999). Age-related synaptic changes in the central nucleus of the inferior colliculus of Fischer-344 rats. *J. Comp. Neurol.* 406 285–298. 10102497

[B25] Hökfelt T. (2010). Looking at neurotransmitters in the microscope. *Prog. Neurobiol.* 90 101–118. 10.1016/j.pneurobio.2009.10.005 19853008

[B26] Hurley L. M. Pollak G. D. (1999). Serotonin differentially modulates responses to tones and frequency-modulated sweeps in the inferior colliculus. *J. Neurosci.* 19 8071–8082. 10.1523/JNEUROSCI.19-18-08071.1999 10479707 PMC6782459

[B27] Hurley L. M. Pollak G. D. (2001). Serotonin effects on frequency tuning of inferior colliculus neurons. *J. Neurophysiol.* 85 828–842. 10.1152/jn.2001.85.2.828 11160516

[B28] Hurley L. M. Pollak G. D. (2005a). Serotonin shifts first-spike latencies of inferior colliculus neurons. *J. Neurosci.* 25 7876–7886. 10.1523/JNEUROSCI.1178-05.2005 16120790 PMC6725259

[B29] Hurley L. M. Pollak G. D. (2005b). Serotonin modulates responses to species-specific vocalizations in the inferior colliculus. *J. Comp. Physiol. A Neuroethol. Sens. Neural Behav. Physiol.* 191 535–546. 10.1007/s00359-005-0623-y 15830241

[B30] Hurley L. M. Sullivan M. R. (2012). From behavioral context to receptors: Serotonergic modulatory pathways in the IC. *Front. Neural Circ.* 6:58. 10.3389/fncir.2012.00058 22973195 PMC3434355

[B31] Hurley L. M. Thompson A. M. Pollak G. D. (2002). Serotonin in the inferior colliculus. *Hear. Res.* 168 1–11. 10.1016/s0378-5955(02)00365-9 12117504

[B32] Jones E. G. Rockel A. J. (1973). Observations on complex vesicles, neurofilamentous hyperplasia and increased electron density during terminal degeneration in the inferior colliculus. *J. Comp. Neurol.* 147 93–118. 10.1002/cne.901470105 4682185

[B33] Keithley E. M. Ryan A. F. Feldman M. L. (1992). Cochlear degeneration in aged rats of four strains. *Hear. Res.* 59 171–178. 1618708 10.1016/0378-5955(92)90113-2

[B34] Kennedy M. J. Ehlers M. D. (2011). Mechanisms and function of dendritic exocytosis. *Neuron* 69 856–875. 10.1016/j.neuron.2011.02.032 21382547 PMC3073864

[B35] Kim H. Kim J. Lee H. Shin E. Kang H. Jeon J. (2021). Baiap3 regulates depressive behaviors in mice via attenuating dense core vesicle trafficking in subsets of prefrontal cortex neurons. *Neurobiol. Stress* 16:100423. 10.1016/j.ynstr.2021.100423 35028340 PMC8715124

[B36] Klepper A. Herbert H. (1991). Distribution and origin of noradrenergic and serotonergic fibers in the cochlear nucleus and inferior colliculus of the rat. *Brain Res.* 557 190–201. 1747753 10.1016/0006-8993(91)90134-h

[B37] Knipper M. Singer W. Schwabe K. Hagberg G. E. Li Hegner Y. Rüttiger L. (2022). Disturbed balance of inhibitory signaling links hearing loss and cognition. *Front. Neural Circ.* 15:785603. 10.3389/fncir.2021.785603 35069123 PMC8770933

[B38] Koehler C. C. Almassri L. S. Tokar N. Mafi A. M. O’Hara M. J. Young J. W. (2023). Age-related changes of GAD1 mRNA expression in the central inferior colliculus. *Transl. Med. Aging* 70 20–32 10.1016/j.tma.2023.04.001 PMC10727507 38111912

[B39] Kommajosyula S. P. Bartlett E. L. Cai R. Ling L. Caspary D. M. (2021). Corticothalamic projections deliver enhanced responses to medial geniculate body as a function of the temporal reliability of the stimulus. *J. Physiol.* 599 5465–5484. 10.1113/JP282321 34783016 PMC10630908

[B40] Li S. Lu Y. Ding D. Ma Z. Xing X. Hua X. (2020). Fibroblast growth factor 2 contributes to the effect of salidroside on dendritic and synaptic plasticity after cerebral ischemia/reperfusion injury. *Aging* 12 10951–10968. 10.18632/aging.103308 32518214 PMC7346066

[B41] Lin Z. Li Y. Hang Y. Wang C. Liu B. Li J. (2022). Tuning the size of large dense-core vesicles and quantal neurotransmitter release via secretogranin II liquid-liquid phase separation. *Adv. Sci.* 9:e2202263. 10.1002/advs.202202263 35896896 PMC9507364

[B42] Lipka J. Kapitein L. C. Jaworski J. Hoogenraad C. C. (2016). Microtubule-binding protein doublecortin-like kinase 1 (DCLK1) guides kinesin-3-mediated cargo transport to dendrites. *EMBO J.* 35 302–318. 10.15252/embj.201592929 26758546 PMC4741305

[B43] Lipman R. D. (1997). Pathobiology of aging rodents: Inbred and hybrid models. *Exp. Gerontol.* 32 215–228. 10.1016/s0531-5565(96)00037-x 9088918

[B44] Lipman R. D. Chrisp C. E. Hazzard D. G. Bronson R. T. (1996). Pathologic characterization of brown Norway, brown Norway Fischer 344, and Fischer 344 brown Norway rats with relation to age. *J. Gerontol. A Biol. Sci. Med. Sci.* 51 B54–B59. 8548501 10.1093/gerona/51A.1.B54 PMC7110307

[B45] Mafi A. Tokar N. Russ M. Barat O. Mellott J. (2022). Age-related ultrastructural changes in the lateral cortex of the inferior colliculus. *Neurobiol. Aging* 120 43–59. 36116395 10.1016/j.neurobiolaging.2022.08.007 PMC10276896

[B46] Mafi A. M. Hofer L. N. Russ M. G. Young J. W. Mellott J. G. (2020). The density of perineuronal nets increases with age in the inferior colliculus in the Fischer Brown Norway rat. *Front. Aging Neurosci.* 11:27. 10.3389/fnagi.2020.00027 32116654 PMC7026493

[B47] Mafi A. M. Russ M. G. Hofer L. N. Pham V. Q. Young J. W. Mellott J. G. (2021). Inferior collicular cells that project to the auditory thalamus are increasingly surrounded by perineuronal nets with age. *Neurobiol. Aging* 105 1–15. 10.1016/j.neurobiolaging.2021.04.001 34004491 PMC8338758

[B48] Malmierca M. S. Izquierdo M. A. Cristaudo S. Hernández O. Pérez-González D. Covey E. (2008). A discontinuous tonotopic organization in the inferior colliculus of the rat. *J. Neurosci.* 28 4767–4776. 10.1523/JNEUROSCI.0238-08.2008 18448653 PMC2440588

[B49] Mastronarde D. (2003). SerialEM: A program for automated tilt series acquisition on tecnai microscopes using prediction of specimen position. *Microsc. Microanal.* 9 1182–1183. 10.1017/S1431927603445911

[B50] Matsuda N. Lu H. Fukata Y. Noritake J. Gao H. Mukherjee S. (2009). Differential activity-dependent secretion of brain-derived neurotrophic factor from axon and dendrite. *J. Neurosci.* 29 14185–14198. 19906967 10.1523/JNEUROSCI.1863-09.2009 PMC3849773

[B51] Mellott J. G. Bickford M. E. Schofield B. R. (2014). Descending projections from auditory cortex to excitatory and inhibitory cells in the nucleus of the brachium of the inferior colliculus. *Front. Syst. Neurosci.* 8:188. 10.3389/fnsys.2014.00188 25339870 PMC4186273

[B52] Meltser I. Canlon B. (2010). The expression of mitogen-activated protein kinases and brain-derived neurotrophic factor in inferior colliculi after acoustic trauma. *Neurobiol. Disease* 40 325–330. 10.1016/j.nbd.2010.06.006 20598895

[B53] Merchán M. Aguilar L. A. Lopez-Poveda E. A. Malmierca M. S. (2005). The inferior colliculus of the rat: Quantitative immunocytochemical study of GABA and glycine. *Neuroscience* 136 907–925. 16344160 10.1016/j.neuroscience.2004.12.030

[B54] Merighi A. (2018). Costorage of high molecular weight neurotransmitters in large dense core vesicles of mammalian neurons. *Front. Cell. Neurosci.* 12:272. 10.3389/fncel.2018.00272 30186121 PMC6110924

[B55] Michael D. J. Cai H. Xiong W. Ouyang J. Chow R. H. (2006). Mechanisms of peptide hormone secretion. *Trends Endocrinol. Metab.* 17 408–415. 17084640 10.1016/j.tem.2006.10.011

[B56] Motts S. D. Schofield B. R. (2009). Sources of cholinergic input to the inferior colliculus. *Neuroscience* 160 103–114. 19281878 10.1016/j.neuroscience.2009.02.036 PMC2700879

[B57] Nakamoto K. T. Mellott J. G. Killius J. Storey-Workley M. E. Sowick C. S. Schofield B. R. (2013). Analysis of excitatory synapses in the guinea pig inferior colliculus: A study using electron microscopy and GABA immunocytochemistry. *Neuroscience* 237 170–183. 10.1016/j.neuroscience.2013.01.061 23395860 PMC3657712

[B58] Nakamoto K. T. Mellott J. G. Killius J. Storey-Workley M. E. Sowick C. S. Schofield B. R. (2014). Ultrastructural characterization of GABAergic and excitatory synapses in the inferior colliculus. *Front. Neuroanat.* 8:108. 10.3389/fnana.2014.00108 25400551 PMC4212260

[B59] Noftz W. A. Beebe N. L. Mellott J. G. Schofield B. R. (2020). Cholinergic projections from the pedunculopontine tegmental nucleus contact excitatory and inhibitory neurons in the inferior colliculus. *Front. Neural Circ.* 14:43. 10.3389/fncir.2020.00043 32765226 PMC7378781

[B60] Noftz W. A. Beebe N. L. Mellott J. G. Schofield B. R. (2021). Dense cholinergic projections to auditory and multisensory nuclei of the intercollicular midbrain. *Hear. Res.* 411:108352. 10.1016/j.heares.2021.108352 34564033 PMC8568689

[B61] Norena A. J. (2011). An integrative model of tinnitus based on a central gain controlling neural sensitivity. *Neurosci. Biobehav. Rev.* 35 1089–1109. 21094182 10.1016/j.neubiorev.2010.11.003

[B62] Oliver D. L. (2005). “Neuronal organization in the inferior colliculus,” in *The Inferior Colliculus*, eds Winer J. Schreiner C. (New York, NY: Springer), 69–114.

[B63] Oliver D. L. Morest D. K. (1984). The central nucleus of the inferior colliculus in the cat. *J. Comp. Neurol.* 222 237–264. 10.1002/cne.902220207 6699209

[B64] Pain S. Brot S. Gaillard A. (2022). Neuroprotective effects of neuropeptide Y against neurodegenerative disease. *Curr. Neuropharmacol.* 20 1717–1725. 10.2174/1570159X19666210906120302 34488599 PMC9881060

[B65] Paloff A. M. Usunoff K. G. (1992). The fine structure of the inferior colliculus in the cat II. synaptic organization. *J. Hirnforsch.* 33 77–106. 1447517

[B66] Paloff A. M. Usunoff K. G. (2000). Tyrosine hydroxylase-like immunoreactive synaptic boutons in the inferior colliculus of the cat. *Ann. Anat.* 182 423–426. 10.1016/S0940-9602(00)80047-3 11035636

[B67] Paloff A. M. Usunoff K. G. Hinova-Palova D. V. Ivanov D. P. (1989). The fine structure of the inferior colliculus in the cat. I. Neuronal perikarya in the central nucleus. *J. Hirnforschung* 30 69–90. 2723415

[B68] Palombi P. S. Caspary D. M. (1996). GABA inputs control discharge rate primarily within frequency receptive fields of inferior colliculus neurons. *J. Neurophysiol.* 75 2211–2219. 10.1152/jn.1996.75.6.2211 8793735

[B69] Parthasarathy A. Bartlett E. L. (2011). Age-related auditory deficits in temporal processing in F-344 rats. *Neuroscience* 192 619–630. 10.1016/j.neuroscience.2011.06.042 21723376

[B70] Parthasarathy A. Herrmann B. Bartlett E. L. (2018). Aging alters envelope representations of speech-like sounds in the inferior colliculus. *Neurobiol. Aging* 73 30–40. 30316050 10.1016/j.neurobiolaging.2018.08.023 PMC6251750

[B71] Paxinos G. Watson C. (1998). *The Rat Brain in Stereotaxic Coordinates.* San Diego, CA: Academic Press.

[B72] Pelletier G. Steinbusch H. W. Verhofstad A. A. (1981). Immunoreactive substance P and serotonin present in the same dense-core vesicles. *Nature* 293 71–72. 10.1038/293071a0 6167867

[B73] Persoon C. M. Moro A. Nassal J. P. Farina M. Broeke J. H. Arora S. (2018). Pool size estimations for dense-core vesicles in mammalian CNS neurons. *EMBO J.* 37:e99672. 10.15252/embj.201899672 30185408 PMC6187028

[B74] Peters A. Palay S. L. Webster H. (1991). in *The Fine Structure of the Nervous System: Neurons and Their Supporting Cells*, ed. Palay S. (Oxford: Oxford University Press).

[B75] R Core Team (2020). *R: A Language and Environment for Statistical Computing.* Vienna: R Foundation for Statistical Computing.

[B76] Ramamoorthy P. Wang Q. Whim M. D. (2011). Cell type-dependent trafficking of neuropeptide Y-containing dense core granules in CNS neurons. *J. Neurosci.* 31 14783–14788. 10.1523/JNEUROSCI.2933-11.2011 21994394 PMC3342306

[B77] Regehr W. G. Carey M. R. Best A. R. (2009). Activity-dependent regulation of synapses by retrograde messengers. *Neuron* 63 154–170. 19640475 10.1016/j.neuron.2009.06.021 PMC3251517

[B78] Reynolds E. (1963). The use of lead citrate at high pH as an electron-opaque stain in electron microscopy. *J. Cell Biol.* 17 208–212. 13986422 10.1083/jcb.17.1.208 PMC2106263

[B79] Ribak C. E. Roberts R. C. (1986). The ultrastructure of the central nucleus of the inferior colliculus of the Sprague-Dawley rat. *J. Neurocytol.* 15 421–438. 10.1007/BF01611726 3746353

[B80] Richardson B. D. Brozoski T. J. Ling L. L. Caspary D. M. (2012). Targeting inhibitory neurotransmission in tinnitus. *Brain Res.* 1485 77–87. 22405692 10.1016/j.brainres.2012.02.014 PMC3374875

[B81] Roberts R. C. Ribak C. E. (1987). An electron microscopic study of GABAergic neurons and terminals in the central nucleus of the inferior colliculus of the rat. *J. Neurocytol.* 16 333–345. 10.1007/BF01611345 3302119

[B82] Robinson L. C. Barat O. Mellott J. G. (2019). GABAergic and glutamatergic cells in the inferior colliculus dynamically express the GABA_*A*_R γ_1_ subunit during aging. *Neurobiol. Aging* 80 99–110. 10.1016/j.neurobiolaging.2019.04.007 31112831

[B83] Rockel A. Jones E. (1973). Observations on the fine structure of the central nucleus of the inferior colliculus of the cat. *J. Comp. Neurol.* 147 61–92. 4682184 10.1002/cne.901470104

[B84] Rumschlag J. A. McClaskey C. M. Dias J. W. Kerouac L. B. Noble K. V. Panganiban C. (2022). Age-related central gain with degraded neural synchrony in the auditory brainstem of mice and humans. *Neurobiol. Aging* 115 50–59. 10.1016/j.neurobiolaging.2022.03.014 35468552 PMC9153923

[B85] Sato T. Wilson T. S. Hughes L. F. Konrad H. R. Nakayama M. Helfert R. H. (2001). Age-related changes in levels of tyrosine kinase B receptor and fibroblast growth factor receptor 2 in the rat inferior colliculus: Implications for neural senescence. *Neuroscience* 103 695–702. 10.1016/s0306-4522(01)00022-7 11274788

[B86] Schneider C. Rasband W. Eliceiri K. (2012). NIH Image to ImageJ: 25 years of image analysis. *Nat. Methods* 9 671–675. 10.1038/nmeth.2089 22930834 PMC5554542

[B87] Schofield B. R. Hurley L. (2018). “Circuits for modulation of auditory function,” in *The Mammalian Auditory Pathways Springer Handbook of Auditory Research*, eds Oliver D. L. Cant N. B. Fay R. R. Popper A. N. (Cham: Springer International Publishing), 235–267.

[B88] Sharma V. Nag T. C. Wadhwa S. Roy T. S. (2009). Temporal distribution of mRNA expression levels of various genes in the developing human inferior colliculus. *Neurosci. Lett.* 461 229–234. 10.1016/j.neulet.2009.06.049 19545602

[B89] Shim H. J. Lee L. H. Huh Y. Lee S. Y. Yeo S. G. (2012). Age-related changes in the expression of NMDA, serotonin, and GAD in the central auditory system of the rat. *Acta Oto-laryngol.* 132 44–50. 10.3109/00016489.2011.622785 22054020

[B90] Silveira M. A. Anair J. D. Beebe N. L. Mirjalili P. Schofield B. R. Roberts M. T. (2020). Neuropeptide Y expression defines a novel class of GABAergic projection neuron in the inferior colliculus. *J. Neurosci.* 40 4685–4699. 10.1523/JNEUROSCI.0420-20.2020 32376782 PMC7294802

[B91] Silveira M. A. Drotos A. C. Pirrone T. M. Versalle T. S. Bock A. Roberts M. T. (2023). Neuropeptide Y signaling regulates recurrent excitation in the auditory midbrain. *J. Neurosci.* 43 7626–7641. 10.1523/JNEUROSCI.0900-23.2023 37704372 PMC10634549

[B92] Singer W. Panford-Walsh R. Knipper M. (2014). The function of BDNF in the adult auditory system. *Neuropharmacology* 76(Pt C), 719–728. 10.1016/j.neuropharm.2013.05.008 23688926

[B93] Sorra K. E. Mishra A. Kirov S. A. Harris K. M. (2006). Dense core vesicles resemble active-zone transport vesicles and are diminished following synaptogenesis in mature hippocampal slices. *Neuroscience* 141 2097–2106. 10.1016/j.neuroscience.2006.05.033 16797135

[B94] Subirana I. Sanz H. Vila J. (2014). Building bivariate tables: The compareGroups Package for R. *J. Stat. Softw.* 57 1–16. 10.18637/jss.v057.i1225400517

[B95] Syka J. (2020). “Age-related changes in the auditory brainstem and inferior colliculus,” in *Aging and Hearing*, eds Helfer K. S. Bartlett E. L. Popper A. N. Fay R. R. (Switzerland: Springer), 67–96.

[B96] Tadros S. F. D’Souza M. Zettel M. L. Zhu X. Lynch-Erhardt M. Frisina R. D. (2007). Serotonin 2B receptor: Upregulated with age and hearing loss in mouse auditory system. *Neurobiol. Aging* 28 1112–1123. 10.1016/j.neurobiolaging.2006.05.021 16822592

[B97] Tao C. L. Liu Y. T. Zhou Z. H. Lau P. M. Bi G. Q. (2018). Accumulation of dense core vesicles in hippocampal synapses following chronic inactivity. *Front. Neuroanat.* 12:48. 10.3389/fnana.2018.00048 29942253 PMC6004418

[B98] Trueta C. De-Miguel F. F. (2012). Extrasynaptic exocytosis and its mechanisms: A source of molecules mediating volume transmission in the nervous system. *Front. Physiol.* 3:319. 10.3389/fphys.2012.00319 22969726 PMC3432928

[B99] Walton J. P. Frisina R. D. O’Neill W. E. (1998). Age-related alteration in processing of temporal sound features in the auditory midbrain of the CBA mouse. *J. Neurosci.* 18 2764–2776. 9502833 10.1523/JNEUROSCI.18-07-02764.1998 PMC6793092

[B100] Walton J. P. Simon H. Frisina R. D. (2002). Age-related alterations in the neural coding of envelope periodicities. *J. Neurophysiol.* 88 565–578. 10.1152/jn.2002.88.2.565 12163510

[B101] Wenstrup J. J. (2005). “The tectothalamic system,” in *The Inferior Colliculus*, eds Winer J. A. Schreiner C. E. (New York, NY: Springer), 200–230. 10.1007/0-387-27083-3_7

[B102] Wissel K. Wefstaedt P. Miller J. M. Lenarz T. Stöver T. (2006). Differential brain-derived neurotrophic factor and transforming growth factor-beta expression in the rat cochlea following deafness. *Neuroreport* 17 1297–1301. 10.1097/01.wnr.0000233088.92839.23 16951573

[B103] Xia X. Lessmann V. Martin T. F. (2009). Imaging of evoked dense-core-vesicle exocytosis in hippocampal neurons reveals long latencies and kiss and- run fusion events. *J. Cell Sci.* 122 75–82. 19066284 10.1242/jcs.034603 PMC2714402

[B104] Yi M. Li H. Wu Z. Yan J. Liu Q. Ou C. (2018). A promising therapeutic target for metabolic diseases: Neuropeptide Y receptors in humans. *Cell Physiol. Biochem.* 1 88–107. 10.1159/000486225 29310113

[B105] Zheng Y. Wildonger J. Ye B. Zhang Y. Kita A. Younger S. H. (2008). Dynein is required for polarized dendritic transport and uniform microtubule orientation in axons. *Nat. Cell Biol.* 10 1172–1180. 10.1038/ncb1777 18758451 PMC2588425

